# Dexmedetomidine inhibits mitochondria damage and apoptosis of enteric glial cells in experimental intestinal ischemia/reperfusion injury via SIRT3-dependent PINK1/HDAC3/p53 pathway

**DOI:** 10.1186/s12967-021-03027-6

**Published:** 2021-11-12

**Authors:** Qin Zhang, Xiao-Ming Liu, Qian Hu, Zheng-Ren Liu, Zhi-Yi Liu, Huai-Gen Zhang, Yuan-Lu Huang, Qiu-Hong Chen, Wen-Xiang Wang, Xue-Kang Zhang

**Affiliations:** 1 Department of Anesthesiology, The First Affiliated Hospital of Nanchang University, No. 17, Yongwaizheng Street, Nanchang, 330006 Jiangxi People’s Republic of China; 2 Department of Thoracic Surgery, The First Affiliated Hospital of Nanchang University, Nanchang, 330006 People’s Republic of China; 3 Department of General Surgery, The First Affiliated Hospital of Nanchang University, Nanchang, 330006 People’s Republic of China

**Keywords:** Intestinal ischemia/reperfusion injury, Dexmedetomidine, Enteric glial cells, Mitochondria damage, Apoptosis

## Abstract

**Background:**

Intestinal ischemia/reperfusion (I/R) injury commonly occurs during perioperative periods, resulting in high morbidity and mortality on a global scale. Dexmedetomidine (Dex) is a selective α2-agonist that is frequently applied during perioperative periods for its analgesia effect; however, its ability to provide protection against intestinal I/R injury and underlying molecular mechanisms remain unclear.

**Methods:**

To fill this gap, the protection of Dex against I/R injury was examined in a rat model of intestinal I/R injury and in an inflammation cell model, which was induced by tumor necrosis factor-alpha (TNF-α) plus interferon-gamma (IFN-γ) stimulation.

**Results:**

Our data demonstrated that Dex had protective effects against intestinal I/R injury in rats. Dex was also found to promote mitophagy and inhibit apoptosis of enteric glial cells (EGCs) in the inflammation cell model. PINK1 downregulated p53 expression by promoting the phosphorylation of HDAC3. Further studies revealed that Dex provided protection against experimentally induced intestinal I/R injury in rats, while enhancing mitophagy, and suppressing apoptosis of EGCs through SIRT3-mediated PINK1/HDAC3/p53 pathway in the inflammation cell model.

**Conclusion:**

Hence, these findings provide evidence supporting the protective effect of Dex against intestinal I/R injury and its underlying mechanism involving the SIRT3/PINK1/HDAC3/p53 axis.

**Supplementary Information:**

The online version contains supplementary material available at 10.1186/s12967-021-03027-6.

## Background

Ischemia–reperfusion (I/R) injury reportedly occurs as a result of interruption of organ blood flow and subsequent restoration; this pathophysiology also accounts for other pathologies including stroke, myocardial infarction, renal I/R injury, intestinal I/R injury and liver I/R injury, ultimately making it a cause of disability or mortality in severe cases [1]. Peculiarly, the intestine is highly sensitive to I/R injury. Intestinal I/R can lead to local tissue damage and destruction of the intestinal mucosal barrier, resulting in the translocation of viable bacteria and endotoxins from the gastrointestinal tract into the distal organs [2, 3]. Moreover, intestinal I/R injury has been found to cause brain damage and memory dysfunction through the activation of microglia, which results in the promotion of neuronal cell apoptosis and inflammatory response [4]. However, the limitations that exist regarding the pathophysiology of intestinal I/R injury has made the development of effective methods for prevention and treatment extremely challenging [5]. The role of dexmedetomidine (Dex) in the treatment of intestinal I/R injury has been highlighted in recent studies, which could be a potential agent for intestinal I/R injury prevention [6].

Dex is an α2-adrenergic receptor agonist that possesses anxiolytic, analgesic, and sedative potentials with minimal suppression of respiratory function [7]. Nonetheless, Dex has been shown to induce low incidence and severity of adverse safety events in infants who received cardiac surgery with cardiopulmonary bypass [8]. Dex has the potential to prevent acute kidney injury by inhibiting mitochondrial damage and cellular inflammation [9]. Dex can inhibit the apoptosis of SH-SY5Y cells by reducing loss of mitochondrial membrane potential and reactive oxygen species, indicating the promising of Dex as a potential drug for the treatment of Parkinson's disease [10]. In addition, Dex has been indicated to exhibit a supportive role in the protection of I/R injury [11, 12]. Dex can be applied for the treatment of I/R; however, its molecular mechanisms are yet to be thoroughly understood [13].

Dex has been proven to reduce lipopolysaccharide-induced acute lung injury via inhibition of mitochondrial dysfunction and cell apoptosis [14]. Additionally, Dex restrains cell apoptosis and phosphatase and tensin homolog (PTEN)-induced putative kinase 1 (PINK1)-medicated mitophagy in lipopolysaccharide-stimulated macrophage injury [15]. Moreover, PINK1 (a nuclear-encoded mitochondrial-targeted kinase) is degraded after a mitochondrial introduction via mitochondrial membrane potential [16]. Additionally, PINK1 kinase also promotes selective autophagy in damaged mitochondria through activation of E3 ubiquitin ligase Parkin [17]. Upregulation of PINK1/Parkin achieved by Sirtuin 3 (SIRT3) activation exerts cardioprotection in cardiac tissues obtained from I/R-exposed rats [18]. SIRT3 is a mitochondrial nicotinamide adenine dinucleotide (NAD)-dependent deacetylase that has also been found to play a crucial role in modulating the mitochondrial function and biosynthetic pathways including oxidative stress, apoptosis, glucose, and fatty acid metabolism [12]. Accumulating studies have further demonstrated the potential role played by SIRT3 in I/R injury treatment [19, 20].

Furthermore, phosphorylation of histone deacetylase 3 (HDAC3) mediated by PINK1 reportedly increased the binding between PINK1 and p53, which subsequently results in hypoacetylation of p53. Additionally, PINK1 knockout leads to partial reduction of HDAC3 expression [21] and this reduction has been demonstrated to exhibit a critical role in I/R injury alleviation [22]. A previous study has indicated that HDAC3 can inhibit the activity of p53 [23]. p53 is a well-established tumor suppressor protein, which regulates cell growth by promoting apoptosis and DNA repair under stressful conditions, but upon mutation, p53 loses its function, inducing abnormal cell proliferation and tumor progression [24]. Notably, p53 inhibition aids the prevention of rat intestinal I/R injury [25]. Herein, with the aim to identify the mechanistic basis behind the protective role of Dex in a rat model of intestinal I/R injury and an inflammation cell model induced by tumor necrosis factor-alpha (TNF-α) plus interferon-gamma (IFN-γ).

## Materials and methods

### Ethics statement

Animal experiments were conducted under the approval of the Institutional Animal Care and Use Committee of the First Affiliated Hospital of Nanchang University, and in strict accordance with the Guide for the Care and Use of Laboratory Animals published by the US National Institutes of Health. All efforts were made to minimize the number and suffering of the included animals.

### Animals

Adult healthy male Sprague–Dawley (SD) rats [specific pathogen-free (SPF) grade; aged 7–9 weeks, weighing about 210–300 g] were purchased from the Department of Pharmacology, Institute of Materia Medica, Chinese Academy of Medical Sciences (Beijing, China). Ten rats received sham operation, 10 were subjected to I/R modeling without other treatment, 10 with I/R injury were pre-treated with 2.5 μg/(kg/h) Dex (Dex1), and 10 with I/R injury were pre-treated with 5 μg/(kg/h) Dex (Dex2). Rats were acclimated for 1 week with room temperature at 23 ± 2 °C and free access to food and water, under a 12-h light cycle. The rats underwent fasting for 12 h before the operation.

### A rat model of intestinal I/R injury

The rats were injected with 1% pentobarbital sodium (30 mg/kg) for anesthesia and were fixed on an operating table in a supine position followed by disinfection with povidone–iodine. A 1–1.5 cm incision was made along the midline of the abdomen. The superior mesenteric artery (SMA) was exposed and clamped with a noninvasive microvascular artery clip to block blood flow. The induction of intestinal ischemia was validated once the following changes were observed: the small intestine appeared to have dark red color, the mesenteric artery ceased to pulsate, and intestinal peristalsis disappeared. The ischemia procedure was continued for 90 min, after which the abdominal cavity was re-opened. The arterial clip was loosened to restore blood supply and allow reperfusion of the intestine. The change of color from dark red to bright red in the small intestine tissues, return of pulsation in the mesenteric artery, and resumption of intestinal peristalsis were indicative of successful reperfusion. Thereafter, the rats were injected with 5% cefoperazone injection (50 mg/kg) to prevent further infection and the incision was closed.

For sham operation, the rats were anaesthetized and continuously infused with 0.9% sodium chloride via tail vein at a rate of 1.5 mL/h for 1 h. The abdomen was then cut open to expose the intestine while the SMA remained unclipped. For I/R model establishment, prior to ischemia induction, the rats were continuously infused with 0.9% sodium chloride via tail vein at a rate of 1.5 mL/h for 1 h. The abdomen was opened to expose the intestine and SMA was clamped for 60 min prior to reperfusion which continued for 120 min [26]. For the Dex1 rat model: before ischemia, hydrochloride 2.5 μg/(kg/h) Dex was continuously infused into rats via a tail vein at a rate of 2.5 μg/kg/h for 1 h followed by the treatment of intestinal I/R injury. The Dex2 rat model was established as follows: before ischemia, hydrochloride 5 μg/(kg/h) Dex was continuously infused into rats via a tail vein at a rate of 5 μg/kg/h for 1 h followed by intestinal I/R treatment. After the experiment, the intestines were collected, fixed with 10% neutral formaldehyde, and paraffin-embedded before morphological analysis. In addition, the intestinal mucosa was washed with normal saline and stored at − 80 °C for subsequent experiments [27, 28].

### Enzyme-linked immunosorbent assay (ELISA)

Monoclonal antibody against Endocan was coated onto a 96-well microplate according to the instructions of the ELISA kit followed by incubation at 4 °C overnight, blocked at room temperature for 1 h, and washed with phosphate-buffered saline (PBS), respectively. The subsequent procedures were conducted as per the kit instructions. The optical density (OD) value at a wavelength of 450 nm was measured [29].

### Measurement of intestinal tissue wet/dry weight (W/D) ratio

After isolated and washed with physiological saline, intestinal tissues were weighed as wet weight. Intestinal tissues were placed in an 80 °C dry box for 24 h, and weighed as dry weight. The intestinal tissue W/D ratio was subsequently calculated [30].

### Hematoxylin–eosin (HE) staining

Tissue specimens were fixed in 4% paraformaldehyde for 30–50 min. After dewaxing and hydrating, the paraffin-embedded sections were stained with hematoxylin for 5 min, and then differentiated with 1% hydrochloric acid, and stained with eosin for 3 min. The sections were then dehydrated, permeabilized, mounted, and observed under a microscope. The intestinal mucosal injury was evaluated by Chiu's classification: 0 point, normal intestinal mucosal villi; 1 point, intestinal mucosa subepithelial space existed in the villi axis, often accompanied by capillary congestion; 2 points, elevation of intestinal mucosal epithelial from the intrinsic membrane and expansion of intestinal subepithelial space; 3 points, elevation of large intestinal mucosal epithelium, with fallen villi to the sides and shed villi tips; 4 points, fallen villi and lamina propria, dilated exposed capillaries, and increased composition of lamina propria cells; 5 points, disintegration or digestion of lamina propria, bleeding, or ulcer formation [31].

### Immunohistochemistry (IHC)

Paraffin sections of clinical tissue specimens were dewaxed and hydrated. The sections were incubated with 3% methanol H_2_O_2_ for 20 min. Antigens were then retrieved and sections were then subjected to goat serum blocking (C-0005, Shanghai Haoran Biotechnology Co., Ltd., Shanghai, China). Sections were probed with rabbit anti-human antibodies to autoimmune glial fibrillary acidic protein (GFAP) (ab7260, 1:200, Abcam, Cambridge, UK) and S-100β (ab52642, 1:200, Abcam, Cambridge, UK) overnight at 4 °C. Next, the sections were incubated with secondary antibody IgG (ab6785, 1:1000, Abcam, Cambridge, UK) at 37 °C for 20 min, followed by the incubation with Streptomyces ovalbumin working solution labeled with horseradish peroxidase (HRP) (0343-10000U; Imunbio Biotechnology Co., Ltd., Beijing, China) at 37 °C for 20 min. Sections were then incubated with diaminobenzidine (DAB; ST033; Guangzhou Weijia Technology Co., Ltd., Guangzhou, China) for color development. Subsequently, sections were counterstained with hematoxylin (PT001; Shanghai Biological Bogu Technology Co., Ltd., Shanghai, China) and underwent blue change of color in 1% ammonia. Sections were dehydrated with gradient alcohol, permeabilized with xylene, and sealed with neutral gum, followed by observation under a microscope. Five high-power microscopic fields were randomly selected from each section, with 100 cells in each field [32].

### Mitochondrial membrane potential (MMP) detection

On the basis of the original grouping, the positive control group was set with the carbonyl cyanide m-chlorophenylhydrazone (CCCP) treatment for evaluation with MMP kit. After 24-h of cell treatment, the cell supernatant was collected and underwent centrifugation. Upon the removal of the supernatant, cells were stained according to the instructions provided on the kit. The normal MMP was detected by the flow cytometry. The Flowjo software was applied to analyze and process the data to obtain the characteristics of cell MMP changes in each group. Each experiment was repeated three times [33].

### Determination of malondialdehyde (MDA) level and superoxide dismutase (SOD) activity

The MDA level and SOD enzyme activity in rat intestinal tissues were assessed by commercially available kits (a003-1 and a001-3-2, NanJing JianCheng Bioengineering Institute, Nanjing, China), respectively. According to the kit instructions, intestinal tissues were made into homogenates and quantified using the bicinchoninic acid (BCA) protein assay. Afterwards, the total MDA level and SOD activity in tissue homogenates were calculated [34].

### TUNEL assay

TUNEL assay was performed to detect apoptosis via In-Situ Cell Death Detection Kit (11684795910, Roche, Basel, Switzerland). Briefly, sections were treated with 0.1% Triton X-100 (Beyotime Institute of Biotechnology, Shanghai, China) at 4 °C for 3 min, and underwent incubation with proteinase K for 15 min, after which it was added with 50 μL of TUNEL working solution. After incubation in a darkroom at 37 °C for 1 h, the plate was sealed with an anti-fluorescence quenching solution and the fluorescence intensity was observed under a fluorescence microscope. Five high-power fields were randomly selected to count the number of TUNEL-positive cells [35].

### Flow cytometry

Cells were digested and washed three times with cold PBS. The precipitate was resuspended with pre-chilled 70% ethanol and allowed to stand at − 20 °C overnight. According to the instructions of Annexin V-fluorescein isothiocyanate (FITC) Apoptosis Detection Kit (K201-100, Biovision, Milpitas, CA, USA), the Annexin-V-FITC, propidium iodide (PI), and *N*-2-hydroxyethyl-piperazine-*N*′-2-ethane sulfonic acid (HEPES) buffer solution was used to prepare Annexin-V-FITC/PI staining solution at a ratio of 1:2:50. Each 100 μL staining solution was used for the re-suspension of 1 × 10^6^ cells, followed by incubation at room temperature for 15 min and the addition of 1 mL of HEPES buffer solution (PB180325, Procell Life Science & Technology, Wuhan, Hubei, China). The apoptosis was evaluated by detecting the fluorescence of FITC and PI at the wavelength of 525 and 620 nm, respectively. Early apoptosis was indicated by the staining of the cells with Annexin-V but not with PI (Annexin-V+/PI−), while cells that were stained with both (Annexin-V+/PI+) were considered to be necrotic or late apoptotic. Cells that were stained with PI alone or (Annexin-V−/PI+) were necrotic [36].

### Transmission electron microscope (TEM)

Intestinal tissues were fixed in 2% glutaraldehyde overnight at 4 °C and fixed with 1% osmium tetroxide (OsO4) for 1 h. Tissues were then dehydrated in ethanol and embedded with epoxy resin. The embedded tissues were sliced using a microtome and the slice was attached to the side of the copper mesh with a supporting film. After negative staining, tissues were observed under a TEM (JEM-1010; JEOL, Tokyo, Japan) [37].

### Cell treatment

Enteric glial cells (EGCs) (American Type Culture Collection, Manassas, VA, USA) were maintained in the RPMI-1640 medium containing 10% fetal bovine serum (FBS; Gibco, Carlsbad, CA, USA), 100 μg/mL penicillin, and 100 μg/mL streptomycin (Gibco, Carlsbad, CA, USA). The medium was placed in an incubator (Thermo Fisher Scientific, Waltham, MA, USA) containing 5% CO_2_ at 37 °C. The untreated EGCs were regarded as controls. As previously described, EGCs were stimulated with 50 ng/mL TNF-α plus 50 ng/mL IFN-γ for approximately 40 h to induce an inflammation cell model [38–40]. The TNF-α- and IFN-γ-stimulated EGCs in the Dex group received treatment with Dex (1 μM) [15]. Further, TNF-α- and IFN-γ-stimulated EGCs in the 3-MA group were treated with 3 mM of 3-Methyladenine (3-MA), a mitophagy inhibitor [41]. Besides, the stimulated cells in the 3-TYP group were treated with 3-(1*H*-1,2,3-triazol-4-yl) pyridine (3-TYP), a well-known inhibitor of SIRT3 [42]. The CCCP treatment was applied for the positive control group, and the HDAC3 inhibitor RGFP966 was used to treat TNF-α- and IFN-γ-stimulated EGCs in the RGFP966 group [43]. TNF-α- and IFN-γ-stimulated EGCs were then transfected with short hairpin RNA (shRNA) targeting PINK1-1 (sh-PINK1-1), sh-PINK1-2, sh-PINK1-3, and their negative control (sh-NC), all purchased from the GenePharma (Shanghai, China), using the Lipofectamine 2000 reagents (Invitrogen, Carlsbad, CA, USA) for 48 h. The oligonucleotides of shRNAs are shown in Additional file 3: Table S1.

### Western blot analysis

Protein was extracted from cells, separated by 10% sodium dodecyl sulfate-polyacrylamide gel electrophoresis (SDS-PAGE) and transferred onto polyvinylidene fluoride (PVDF) membrane. The membrane was then blocked with 5% bovine serum albumin (BSA) for 2 h at room temperature and probed with primary anti-rabbit antibodies (Abcam, Cambridge, UK) to GFAP (ab7260), p53 (ab32389), HDAC3 (ab32369, 1:2000), anti-LC3-II (ab51520, 1:2000), phosphorylated (p)-HDAC3 (ab61056), S-100β (ab52642), PINK1 (ab23707), SIRT3 (ab223531), Bcl-2-associated X protein (Bax) (ab32503), B-cell lymphoma 2 (Bcl-2) (ab32124), caspase-3 (ab197202), cleaved caspase-3 (ab2302, 1:500) and glyceraldehyde-3-phosphate dehydrogenase (GAPDH) (ab181602, 1:500) overnight at 4 °C. The membrane was then re-probed with the HRP-labeled secondary goat anti-rabbit antibody (ab205719, 1:2000, Abcam) for 1 h at room temperature. The membrane was exposed to X-ray film for 5–10 min, followed by visualization, and fixing. ImageJ software was adopted for gray value quantitative analysis. GAPDH served as the internal reference.

### Co-immunoprecipitation (Co-IP)

Cells were lysed in lysis buffer (50 mM Tris–HCl (pH 7.4), 150 mM NaCl, 10% glycerol, 1 mM ethylenediaminetetraacetate (EDTA), 0.5% of 40-ethylene oxide units per molecule (NP-40) and protease inhibitor). The lysate underwent incubation with 1 μg anti-Pan Acetylation (66289-1-Ig; ProteinTech Group, Chicago, IL, USA) by the addition of 15 μL protein A along with G-beads (Santa Cruz Biotechnology, Santa Cruz, CA, USA) for 2 h. The mixture was heated at 100 °C for 5 min and the acetylation level of p53 was determined using Western blot analysis [44].

### Statistical analysis

Data were processed using SPSS 21.0 statistical software (IBM Corp. Armonk, NY, USA). Measurement data were expressed as mean ± standard deviation. Data between two groups were compared using unpaired *t*-test. Comparison among multiple groups was conducted by one-way analysis of variance (ANOVA) with Tukey’s post hoc test. *p* < 0.05 was indicative of statistical significance.

## Results

### Dex treatment reduces oxidative stress and protects rats against experimentally induced intestinal I/R injury

**Fig. 1:**
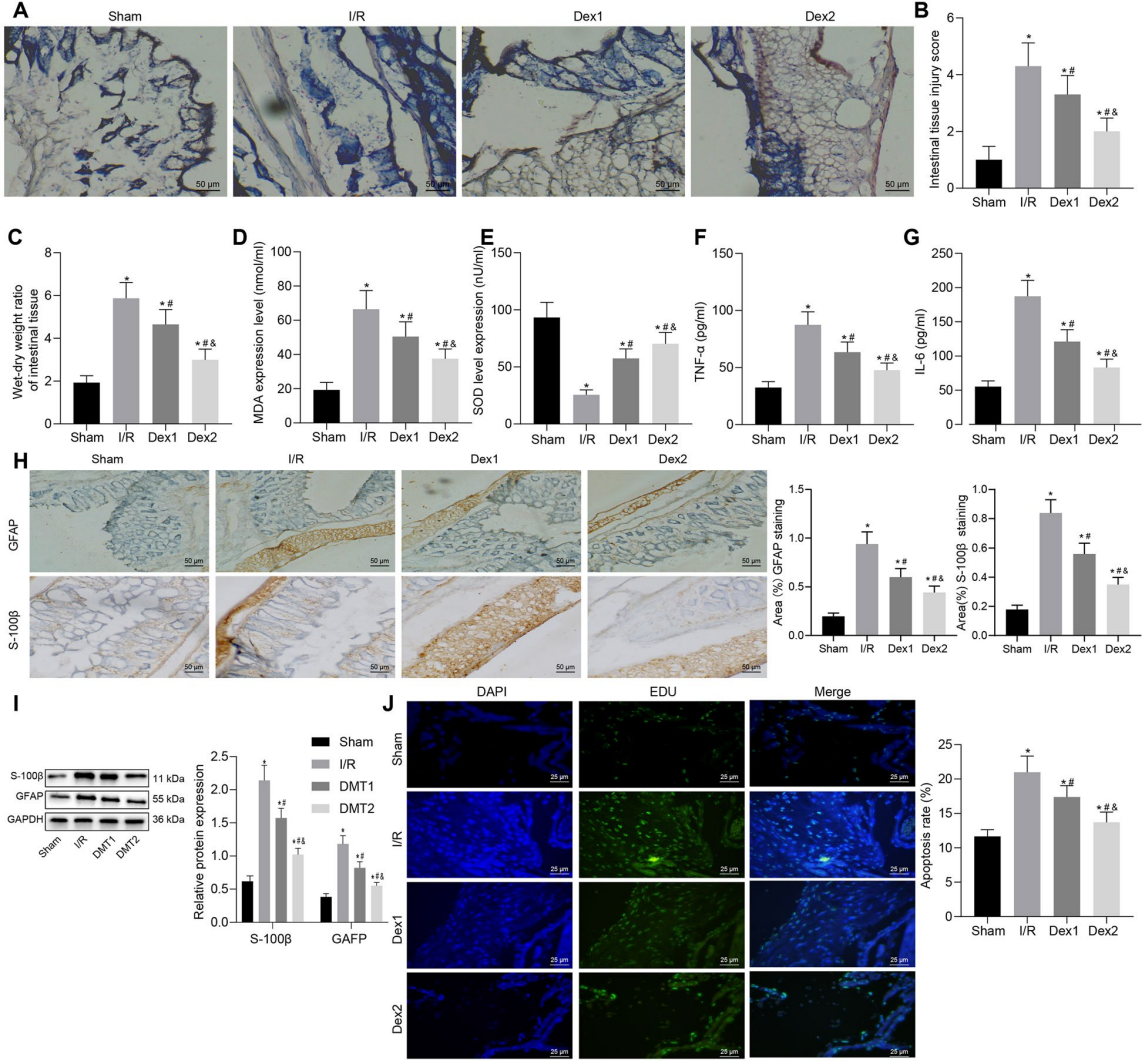
Dex reduces oxidative stress and consequently alleviates intestinal I/R injury in rats. **A** Intestinal histopathological changes in rats. **B** Intestinal damage assessed by Chiu’s score. **C** Intestinal W/D ratio. **D** Level of MDA in intestinal tissues of rats. **E** SOD activity in intestinal tissues of rats. **F** Serum expression of TNF-α in rats. **G** Serum expression of IL-6 in rats. **H** The positive expression of GFAP and S-100β in the intestinal tissue of rats evaluated by IHC. **I** Western blot analysis for determination of protein expression of S-100β and GFAP in intestinal tissues of rats. **J** The apoptosis of EGCs in intestinal tissues of rats examined by TUNEL. *p < 0.05 vs. sham-operated rats; ^#^*p* < 0.05 vs. rats with I/R injury; ^&^*p* < 0.05 vs. rats with Dex1 (2.5 μg/(kg/h) Dex) treatment. Measurement data were expressed as mean ± standard deviation. Comparison among multiple groups was conducted by one-way ANOVA with Tukey’s post hoc test. N = 10

First, we established a rat model of intestinal I/R injury by inducing SMA occlusion followed by injection with Dex before reperfusion. Analysis on the intestinal tissues of rats using HE staining suggested that compared with sham-operated rats, rats following I/R injury exhibited severe intestinal tissue damage accompanied by large number of inflammatory cell infiltration and some visible bleeding. However, Dex1 treatment demonstrated partial degeneration and necrosis of mucosal epithelial cells in intestinal tissues and reduced degree of epithelial cell shedding and inflammatory cell infiltration. The rats with Dex2 treatment were observed to have a partially damaged intestinal mucosa, mild inflammatory cell infiltration, and slightly damaged intestinal villi (Fig. 1A). In addition, Chiu's score results revealed that the intestinal tissue injury scores of rats with I/R injury were increased in comparison with sham-operated rats. Moreover, compared with rats with I/R injury, the scores for intestinal tissue injury were lower in rats treated with Dex1 or Dex2, of which Dex2 led to a further decline of injury score than Dex1 (Fig. 1B). Moreover, rats with I/R injury had higher intestinal W/D ratio than sham-operated rats. In addition, Dex1 or Dex2 treatment triggered a decline in intestinal W/D ratio in. Dex2 treatment resulted in a lower intestinal W/D ratio in comparison with Dex1 treatment (Fig. 1C).

Additionally, the rats with I/R injury presented with decreased SOD activity and elevated levels of MDA in comparison to sham-operated rats. In contrast to rats with I/R injury, rats with Dex1 or Dex2 treatment showed an increase in SOD activity and diminished levels of MDA. The rats with Dex2 treatment displayed a higher SOD activity and lower levels of MDA compared with rats treated with Dex1 treatment (Fig. 1D, E). Moreover, ELISA results revealed an enhancement in the serum levels of TNF-α and IL-6 in rats exposed to I/R injury while decreased serum levels of TNF-α and IL-6 were observed in I/R-exposed rats with Dex1 or Dex2 treatment. Meanwhile, a more pronounced decline in serum levels of TNF-α and IL-6 was noted in rats with Dex2 treatment compared to those with Dex1 treatment (Fig. 1F, G).

The protective mechanism of EGCs on intestinal barrier function has been confirmed previously [45]. It has been indicated that the regulation of EGCs on the structure and function of the intestinal tract relies on its specific protein expression, which mainly includes the GFAP and S-100β [46]. The results of IHC showed the presence of GFAP in the EGCs of rat ileal tissues while the tan particles shown in Fig. 1H were GFAP-positive cells. Moreover, I/R injury rats presented increased positive expression of GFAP and S-100β in comparison to sham-operated rats. The positive expression of GFAP and S-100β in rats treated with Dex1 or Dex2 was decreased compared to those with I/R injury. Dex2 treatment led to a more obvious reduction in the positive expression of GFAP and S-100β than Dex1 treatment (Fig. 1H). Western blot analysis results showed increased protein expression of S-100β and GFAP in I/R injury rat models compared to the sham-operated rats. However, rats with Dex1 or Dex2 treatment displayed decreased protein expression of S-100β and GFAP compared to rats with I/R injury. In contrast to rats with Dex1 treatment, the protein expression of S-100β and GFAP was further reduced in rats with Dex2 treatment (Fig. 1I). TUNEL results suggested enhanced apoptosis rate in rats with I/R injury in comparison to sham-operated rats while opposite results were noted following Dex1 or Dex2 treatment (Fig. 1J). Taken together, the aforementioned results provide evidence that Dex inhibited oxidative stress and thus arrested intestinal I/R injury in rats.

### Dex promotes mitophagy and inhibits apoptosis of TNF-α- and IFN-γ-stimulated EGCs

**Fig. 2:**
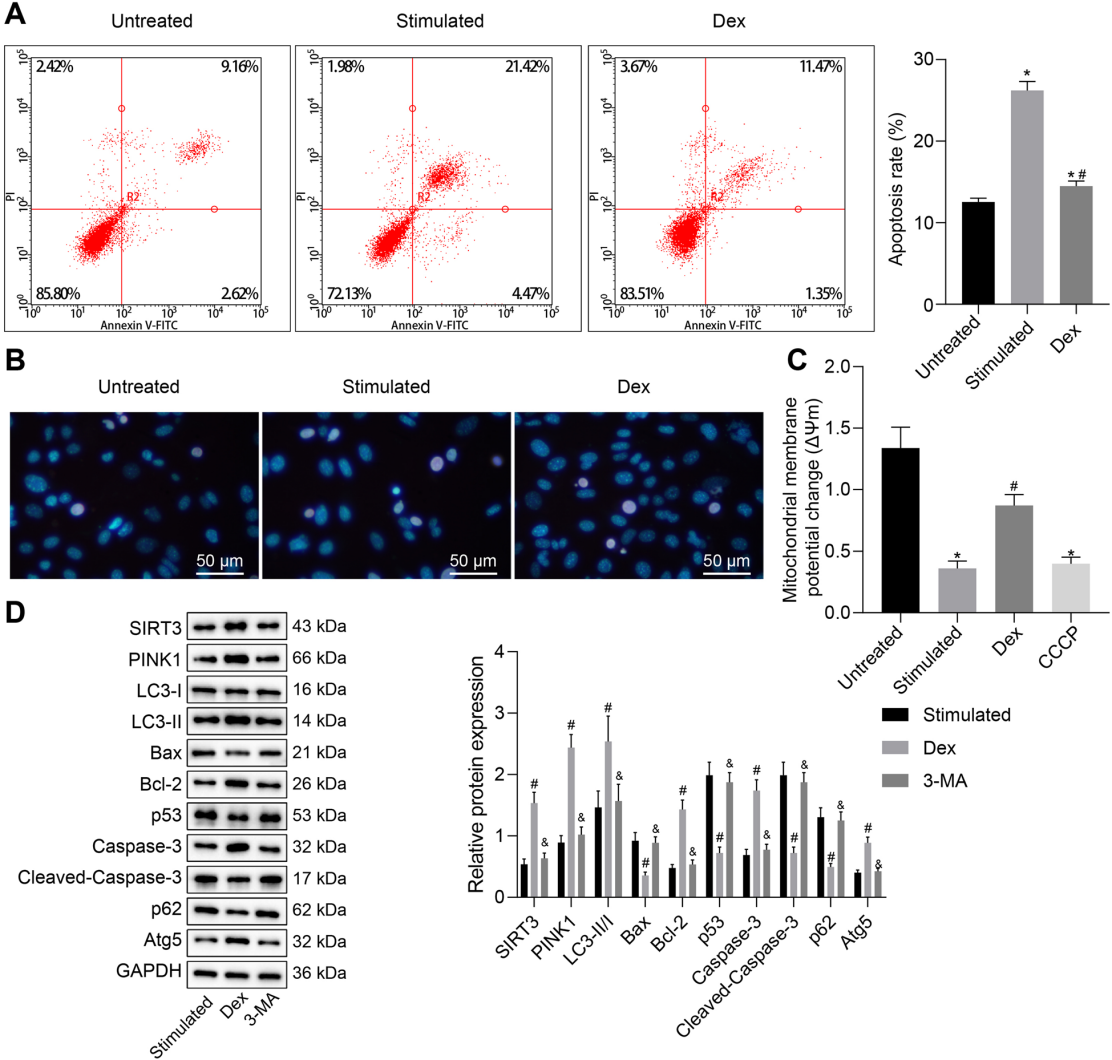
Dex reduces TNF-α- and IFN-γ-stimulated mitochondrial damage and apoptosis of EGCs. **A** Assessment of apoptosis by Annexin-V/PI in EGCs after 48 h of Dex treatment examined by flow cytometry. Cells were considered to be in early apoptosis, that was stained with Annexin-V but not with PI (Annexin-V+/PI−), and cells that were stained with both (Annexin-V+/PI+) were considered to be necrotic or late apoptotic. Cells that were stained with PI alone or (Annexin-V−/PI+) were necrotic. At least 10,000 cells for each sample were analyzed in each experiment. **B** The changes of EGCs morphology observed by Hoechst 33,258 staining. **C** Detection of MMP in EGCs. **D** LC3-II/I ratio and protein expression of Atg5, p62, Bax, Bcl-2, caspase-3, and cleaved caspase-3 in the EGCs measured by Western blot analysis. *p < 0.05 vs. EGCs without any treatment; ^#^*p* < 0.05 vs. EGCs treated with 50 ng/mL of TNF-α, 100 ng/mL of IFN-γ; ^&^*p* < 0.05 vs. Dex group (EGCs treated with 50 ng/mL of TNF-α, 100 ng/mL of IFN-γ and 1 μM of Dex). Measurement data were expressed as mean ± standard deviation. Comparison among multiple groups was conducted by one-way ANOVA with Tukey’s post hoc test. The cell experiment was repeated three times

Next, we shifted our attention to determine the effects of Dex on EGCs in an inflammatory environment. Flow cytometric data suggested that early apoptosis rate of TNF-α- and IFN-γ-stimulated cells was increased in comparison with the control cells. However, increased apoptosis rate induced by TNF-α and IFN-γ was negated by Dex treatment (Fig. 2A). Thereafter, the Hoechst33342 staining results revealed that TNF-α- and IFN-γ-stimulated EGCs showed more prominent apoptotic characteristics as evidenced by decreased number of cells, nuclear condensation, chromatin condensation, and the formation of apoptotic bodies, whereas the apoptotic characteristics were attenuated upon Dex treatment (Fig. 2B). As shown in Fig. 2C, MMP was diminished in TNF-α- and IFN-γ-stimulated or CCCP-treated EGCs, while Dex treatment increased MMP in TNF-α- and IFN-γ-stimulated EGCs.

Existing literature has identified that SIRT induces mitophagy by mediating the PINK1/PRKN signaling pathway [47]. Ischemic postconditioning (IPO) treatment can ameliorate intestinal I/R injury by evoking autophagy, activating Akt, inactivating GSK-3, and activating Nrf2 [48]. Therefore, we then aimed to determine whether Dex affects mitochondrial function and apoptosis by increasing the expression of PINK1/PRKN in the TNF-α- and IFN-γ-stimulated EGCs. Western blot analysis was performed to determine the expression of autophagy-related proteins (LC3-II/I, Atg5 and p62) and apoptosis-related proteins (Bax, Bcl-2, caspase-3, and cleaved caspase-3) in TNF-α- and IFN-γ-stimulated EGCs. The results indicated that Dex treatment resulted in increased LC3-II/I ratio and upregulated levels of SIRT3, PINK1, Bcl-2, caspase-3 and Atg5 concomitant with diminished levels of Bax, p53, p62 and cleaved caspase-3 in TNF-α- and IFN-γ-stimulated EGCs, while additional 3-MA treatment led to opposite results (Fig. 2D). In addition, TEM observation results revealed that Dex treatment led to the development of mitochondrial autophagosomes in TNF-α- and IFN-γ-stimulated EGCs, while autophagosomes disappeared following treatment with additional 3-MA (Additional file 1: Figure S1A). Taken together, the above results demonstrated that Dex promoted mitophagy and MMP and inhibited apoptosis in EGCs under conditions of inflammation.

### Dex prevents mitochondrial damage and apoptosis in TNF-α- and IFN-γ-stimulated EGCs by regulating SIRT3 and PINK1 expression

**Fig. 3:**
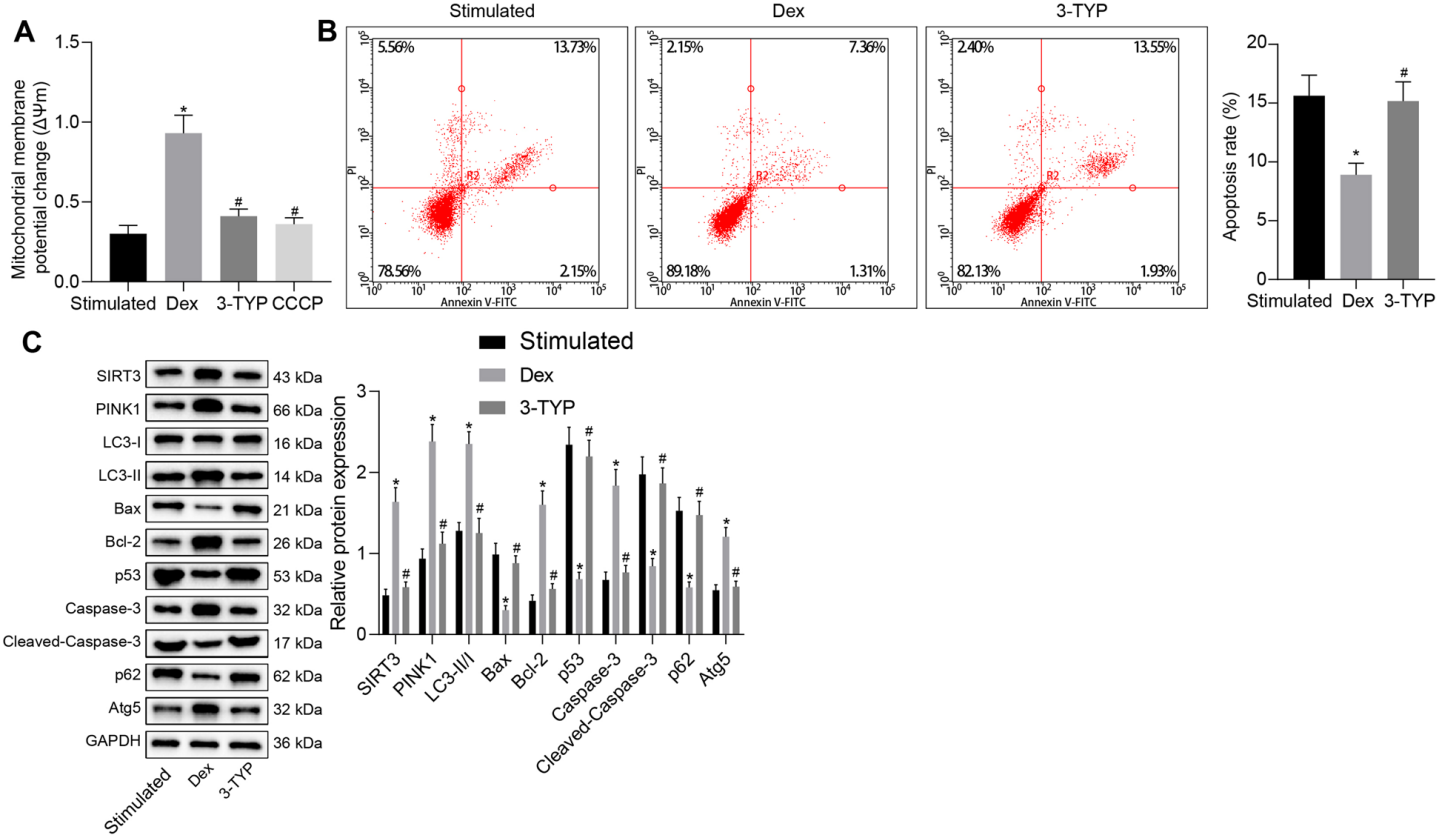
Dex restrains TNF-α- and IFN-γ-stimulated mitochondrial damage and apoptosis in EGCs through the SIRT3-mediated PINK1-related pathway. **A** Detection of MMP changes in EGCs. **B** The apoptosis of EGCs examined by flow cytometry. **C** Protein expression of SIRT3, PINK1, Bax, Bcl-2, Atg5, p62, p53, caspase-3, and cleaved caspase-3 along with LC3-II/I ratio in the EGCs measured by Western blot analysis. *p < 0.05 vs. the Mod group (EGCs treated with 50 ng/mL of TNF-α and 100 ng/mL of IFN-γ); ^#^*p* < 0.05 vs. EGCs treated with 50 ng/mL of TNF-α, 100 ng/mL of IFN-γ and 1 μM of Dex. Measurement data were expressed as mean ± standard deviation. Comparison among multiple groups was conducted by one-way ANOVA with Tukey’s post hoc test. The cell experiment was repeated three times

The following experiments focused at exploring the mechanism of how Dex promotes mitophagy and inhibits apoptosis of EGCs. Figure 3A illustrates an increase of MMP in TNF-α- and IFN-γ-stimulated cells treated with Dex but it was reversed following treatment with 3-TYP, a inhibitor of SIRT3, which was close to CCCP treatment. Moreover, the results of flow cytometry with Annexin V/PI double staining suggested that Dex treatment reduced early apoptosis rate of TNF-α- and IFN-γ-stimulated cells, which was neutralized by the 3-TYP treatment (Fig. 3B). In addition, TNF-α- and IFN-γ-exposed EGCs with Dex treatment demonstrated increased LC3-II/I ratio and elevated levels of SIRT3, PINK1, Bcl-2, Atg5 and caspase-3 as well as decreased levels of Bax, p53, p62 and cleaved caspase-3. However, the effect of Dex was abolished by 3-TYP treatment (Fig. 3C).

Furthermore, TEM observation results showed that EGCs with TNF-α and IFN-γ treatment demonstrated normal mitochondrial morphology. EGCs with Dex treatment showed mitochondrial autophagosomes and boosted mitophagy while those with additional 3-TYP treatment presented a morphological change of mitochondria i.e., mitochondrial swelling, the fractured and finally disappeared cristae (Additional file 1: Figure S1B). Collectively, these results demonstrated that Dex resulted in the suppression of mitochondrial damage and apoptosis of EGCs through the SIRT3-mediated PINK1-related pathway.

### PINK1 reduces p53 expression through promotion of phosphorylation of HDAC3

**Fig. 4:**
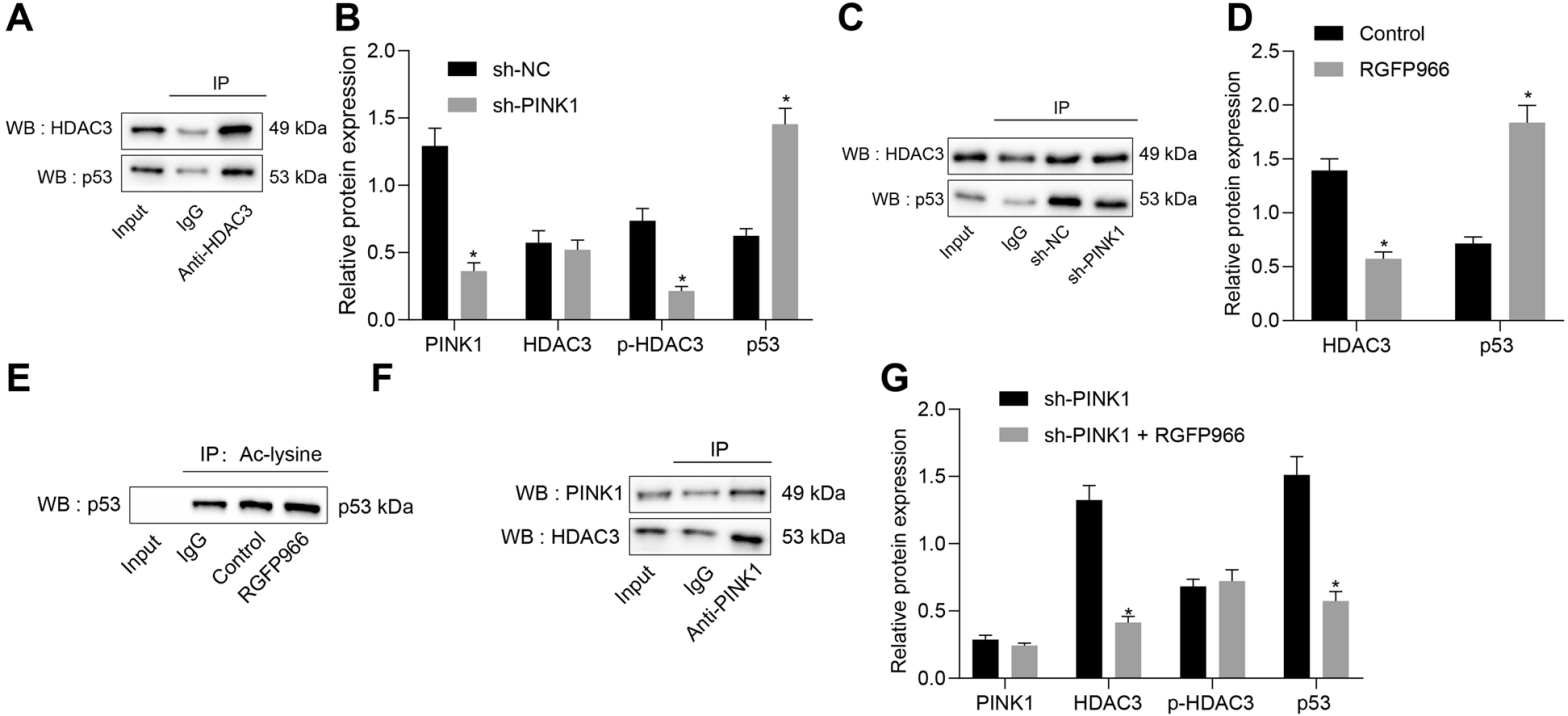
PINK1 reduces p53 expression by promoting the phosphorylation of HDAC3. **A** Binding between HDAC3 and p53 in EGCs verified by Co-IP. **B** HDAC3 phosphorylation level and protein expression of PINK1, HDAC3, and p53 in EGCs transfected with sh-PINK1 determined by Western blot analysis. **C** Interaction among PINK1, HDAC3, and p53 in EGCs transfected with sh-PINK1 identified by Co-IP. **D** Western blot analysis for determination of protein expression of HDAC3 and p53 in EGCs with RGFP966 treatment. **E** The acetylation level of p53 assessed by Co-IP. **F** Binding between HDAC3 and PINK1 in EGCs verified by Co-IP. **G** HDAC3 phosphorylation level and protein expression of PINK1, HDAC3, and p53 in EGCs transfected with sh-PINK1 measured through Western blot analysis. *p < 0.05 vs. EGCs transfected with sh-NC, EGCs treated with 50 ng/mL of TNF-α and 100 ng/mL of IFN-γ) or TNF-α and IFN-γ -exposed EGCs transfected with sh-PINK1. Measurement data were expressed as mean ± standard deviation. Comparison among multiple groups was conducted by one-way ANOVA with Tukey’s post hoc test. *p* < 0.05 was indicative of statistical significance. The cell experiment was repeated three times

Next, we sought to test the downstream mechanism of PINK1 in EGCs. Co-IP assay results indicated the presence of HDAC3 protein in the immune complex (Fig. 4A), thus suggesting the binding between PINK1 and HDAC3 in EGCs. Moreover, western blot analysis results demonstrated that silencing of PINK1 resulted in a reduction of PINK1 expression and HDAC3 phosphorylation level, whereas the expression of p53 was increased (Fig. 4B). The results of Co-IP assay revealed that knockdown of PINK1 weakened the binding between HDAC3 and p53 in EGCs (Fig. 4C).

To further verify the effect of HDAC3 on p53 protein, we used the HDAC3 inhibitor RGFP966 to treat EGCs induced with TNF-α plus IFN-γ. Western blot analysis data revealed that RGFP966 treatment resulted in a significant reduction of HDAC3 expression but elevated the expression of p53 (Fig. 4D). Furthermore, RGFP966 treatment remarkably elevated the acetylation level of p53 (Fig. 4E), indicating that HDAC3 inhibits the acetylation of p53 protein. Co-IP results detected the presence of PINK1 protein in the immune complex (Fig. 4F), suggesting the binding between PINK1 and HDAC3 in EGCs. Moreover, Western blot analysis data revealed that after the induction of TNF-α and IFN-γ, EGCs co-treated with sh-PINK1 and RGFP966 demonstrated reduced expression of HDAC3 and elevated expression of p53 compared with those treated with sh-PINK1 alone (Fig. 4G). Collectively, these results verified the role of PINK1 in promoting the inhibitory role of p53 caused by the HDAC3 phosphorylation.

### PINK1 represses mitochondrial damage and apoptosis in TNF-α and IFN-γ-stimulated EGCs through inhibition of p53

**Fig. 5:**
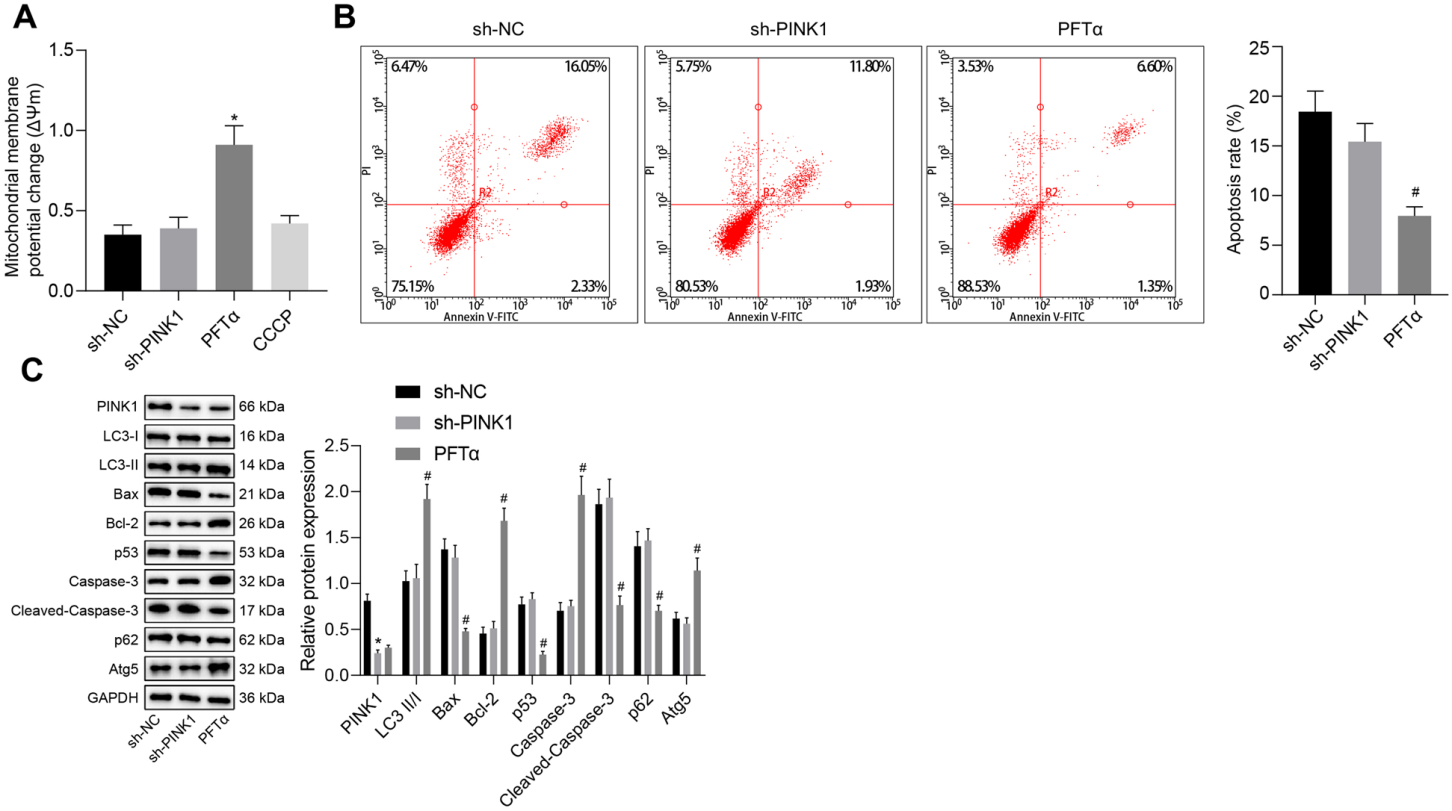
PINK1 suppresses mitochondrial damage and cell apoptosis of TNF-α- and IFN-γ-stimulated EGCs via inhibition of p53. **A** Examination of MMP changes in EGCs. **B** The apoptosis of EGCs detected by flow cytometry. **C** Protein expression of PINK1, Bax, Bcl-2, Atg5, p62, p53, caspase-3, and cleaved caspase-3 along with LC3-II/I ratio in the EGCs determined by Western blot analysis. *p < 0.05 vs. EGCs treated with 50 ng/mL of TNF-α and 100 ng/mL of IFN-γ following with sh-NC transfection; ^#^*p* < 0.05 vs. EGCs treated with 50 ng/mL of TNF-α and 100 ng/mL of IFN-γ following with sh-PINK1 transfection. Measurement data were expressed as mean ± standard deviation. Comparison among multiple groups was conducted by one-way ANOVA with Tukey’s post hoc test. The cell experiment was repeated three times. PFT-α, pifithrin-α

After uncovering that PINK1 inhibited p53 expression by promoting the phosphorylation level of HDAC3, we next aimed to investigate the effect of PINK1 and p53 on the mitochondrial damage and apoptosis of TNF-α and IFN-γ-exposed EGCs. There were no significant changes observed in the MMP between TNF-α and IFN-γ-exposed EGCs transfected with sh-NC and sh-PINK1, while Pifithrin-α treatment resulted in elevated MMP (Fig. 5A). Flow cytometric analysis showed no significant difference in the cell apoptosis between TNF-α and IFN-γ-exposed EGCs with sh-NC transfection and those with sh-PINK1 transfection, whereas the early apoptosis rate was diminished by Pifithrin-α treatment (Fig. 5B). Moreover, after the induction of TNF-α and IFN-γ, EGCs treated with sh-PINK1 presented with poor expression of PINK1. Pifithrin-α treatment resulted in increased LC3-II/I ratio and elevated levels of Bcl-2, Atg5 and caspase-3 as well as reduced levels of Bax, p53, p62 and cleaved caspase-3 (Fig. 5C). Moreover, the results of TEM suggested that the TNF-α and IFN-γ-exposed EGCs with sh-NC or sh-PINK1 transfection presented normal mitochondrial morphology, while Pifithrin-α treatment led to the appearance of mitochondrial autophagosomes and augmented mitophagy (Additional file 1: Figure S1C). Hence, these findings verified that PINK1 inhibits mitochondrial damage and cell apoptosis of EGCs via p53 inhibition.

### Dex suppresses mitochondrial damage and apoptosis of TNF-α and IFN-γ-stimulated EGCs through SIRT3-mediated PINK1/HDAC3/p53 pathway

**Fig. 6:**
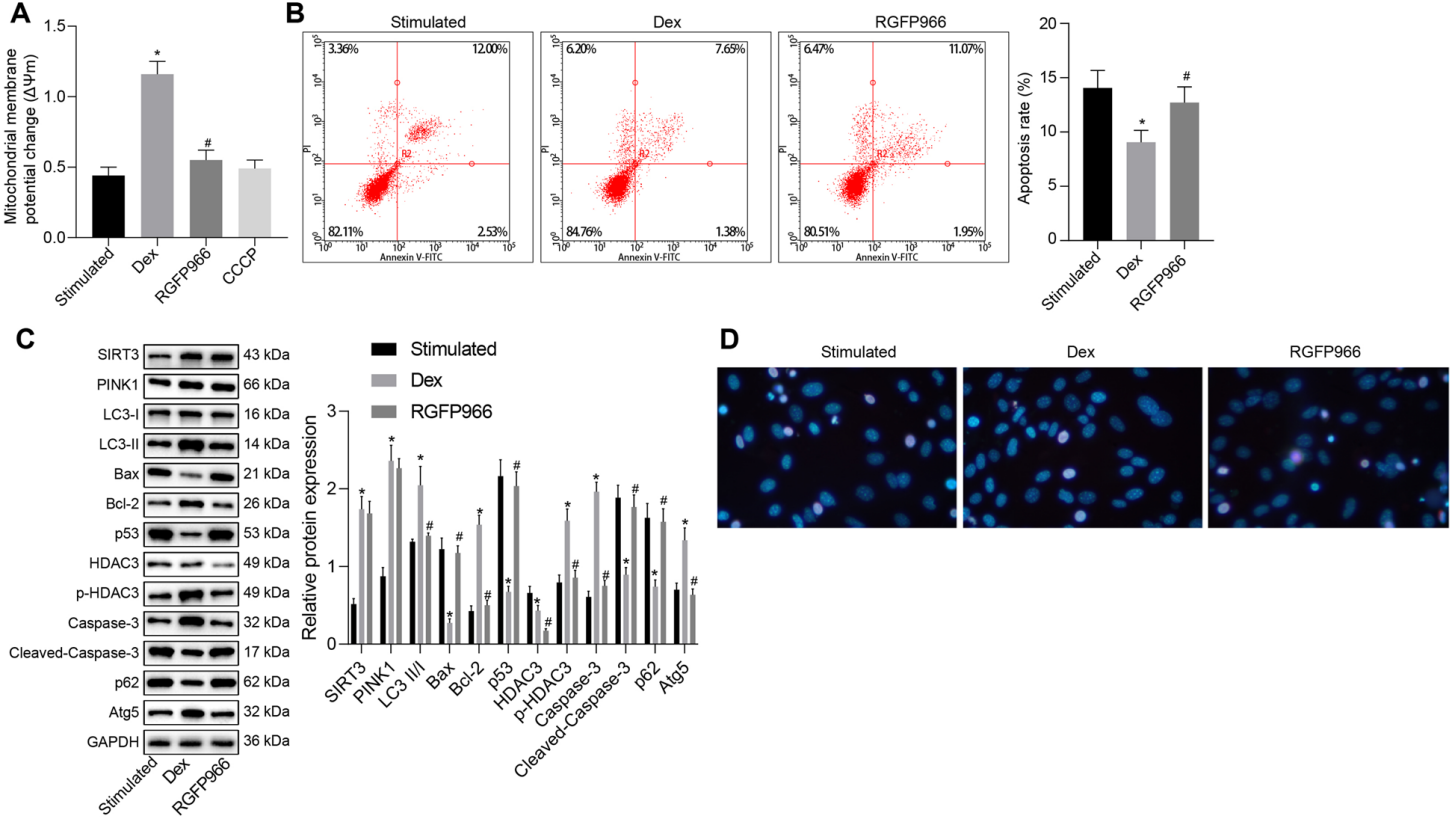
Dex ameliorates TNF-α- and IFN-γ-stimulated mitochondrial damage and apoptosis in EGCs by the SIRT3-mediated PINK1/HDAC3/p53 pathway. **A** Examination of MMP changes in EGCs. **B** The apoptosis of EGCs assessed by flow cytometry. **C** Western blot analysis for measurement of protein expression of SIRT3, PINK1, Bax, Bcl-2, Atg5, p62, p53, caspase-3, and cleaved caspase-3 along with LC3-II/I ratio in the EGCs. **D** Apoptosis of EGCs was detected by Hoechst33342 staining and observed in a fluorescence microscope. *p < 0.05 vs. EGCs treated with 50 ng/mL of TNF-α and 100 ng/mL of IFN-γ; ^#^*p* < 0.05 vs. EGCs treated with 50 ng/mL of TNF-α, 100 ng/mL of IFN-γ and 1 μM of Dex. Measurement data were expressed as mean ± standard deviation. Comparison among multiple groups was conducted by one-way ANOVA with Tukey’s post hoc test. The cell experiment was repeated three times

In order to explore whether Dex can regulate the mitochondrial damage and apoptosis of EGCs through the SIRT3-mediated PINK1/HDAC3/p53 pathway, EGCs were induced with TNF-α and IFN-γ to stimulate the inflammation model followed by treatment with Dex alone or in the presence of 10 μM of RGFP966. Notably, our results showed enhanced MMP after Dex treatment but it was abolished by the additional treatment of RGFP966, which was close to the CCCP treatment (Fig. 6A). Thereafter, flow cytometry reported that in contrast to untreated TNF-α- and IFN-γ-stimulated EGCs, those with Dex treatment were observed to have reduced early apoptosis rate, which was neutralized by the RGFP966 treatment (Fig. 6B).

Furthermore, western blot analysis results showed that TNF-α and IFN-γ-exposed EGCs with Dex treatment displayed increased LC3-II/I ratio and augmented HDAC3 phosphorylation level, and expression of SIRT3, PINK1, Bcl-2, Atg5 and caspase-3, whereas the expression of Bax, HDAC3, p53, p62 and cleaved caspase-3 was reduced, which were reversed by the additional RGFP966 treatment (Fig. 6C). Furthermore, Hoechst33342 staining results of EGC apoptosis demonstrated that Dex treatment resulted in a reduction of EGC apoptosis, which was reversed following treatment with RGFP966 (Fig. 6D). The results from TEM observation manifested that the TNF-α- and IFN-γ-exposed EGCs presented normal mitochondrial morphology, whereas TNF-α- and IFN-γ-stimulated EGCs with Dex treatment showed augmented mitophagy. However, RGFP966 treatment caused a morphological change of mitochondria (Additional file 1: Figure S1D). Hence, these results demonstrated that Dex inhibits mitochondrial damage and cell apoptosis of EGCs through the SIRT3-mediated PINK1-related pathway.

### Dex protects against intestinal I/R injury through SIRT3-mediated PINK1/HDAC3/p53 pathway

**Fig. 7:**
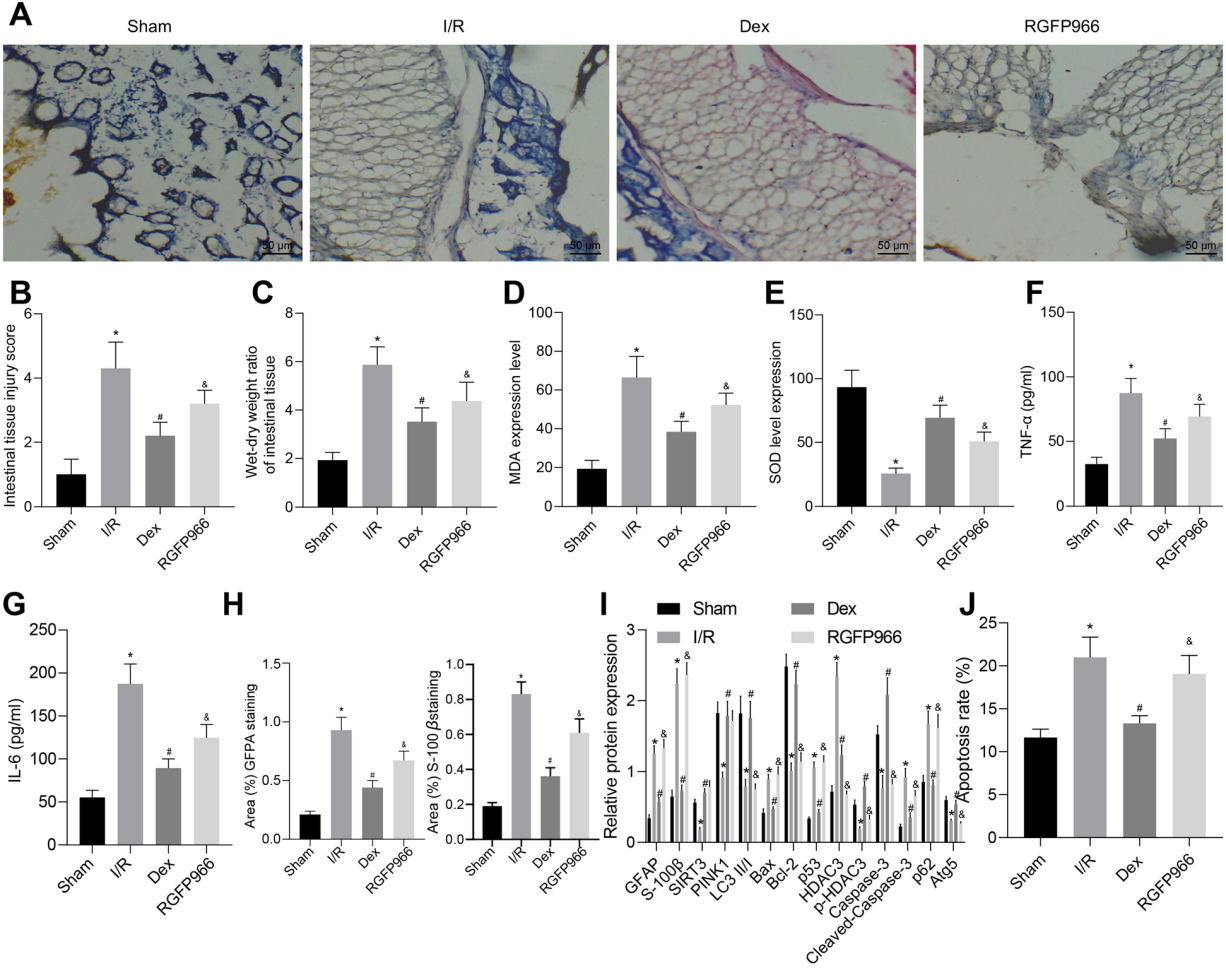
Dex ameliorates intestinal I/R injury in rats through the SIRT3-mediated PINK1/HDAC3/p53 pathway. **A** Intestinal histopathological changes in rats. **B** Intestinal damage assessed by Chiu’s score. **C** Intestinal W/D ratio. **D** Level of MDA in intestinal tissues of rats. **E** SOD activity in intestinal tissues of rats. **F** Serum expression of TNF-α in rat tissues. **G** Serum expression of IL-6 in rat tissues. **H** The positive expression of GFAP and S-100β in the intestinal tissue of rats evaluated by IHC. **I** Western blot analysis for measurement of HDAC3 phosphorylation level and protein expression of GFAP, S-100β, SIRT3, PINK1, Bax, Bcl-2, Atg5, p62, p53, HDAC3, caspase-3, and cleaved caspase-3 as well as LC3-II/I ratio in intestinal tissues of rats. **J** The apoptosis of EGCs in intestinal tissues of rats examined by TUNEL. *p < 0.05 vs. sham-operated rats; ^#^*p* < 0.05 vs. I/R-exposed rats; ^&^*p* < 0.05 vs. I/R-exposed rats with Dex treatment. Measurement data were expressed as mean ± standard deviation. Comparison among multiple groups was conducted by one-way ANOVA with Tukey’s post hoc test. N = 10

Finally, we aimed to characterize the effects of Dex on intestinal I/R injury through the SIRT3-mediated PINK1/HDAC3/p53 pathway in a rat model of intestinal I/R injury. HE staining analysis on intestinal tissues showed severe intestinal tissue damage in I/R-exposed rats while Dex treatment caused partial damaged intestinal mucosa. Additional RGFP966 treatment resulted in partial damaged intestinal mucosa (Fig. 7A). Results of Chiu’s classification and calculation of W/D ratio of intestinal tissues suggested that the intestinal tissue injury scores and W/D ratio of intestinal tissues of I/R-exposed rats were elevated in comparison with sham-operated rats. However, after the induction of I/R, rats treated with Dex showed a decreased score and W/D ratio of intestinal tissues which was negated by treatment with RGFP966 (Fig. 7B, C).

Measurement results of SOD activity and levels of MDA demonstrated declined SOD activity and enhanced levels of MDA in the rats exposed to I/R while Dex treatment led to opposite results. In addition, RGFP966 treatment diminished SOD activity and increased levels of MDA (Fig. 7D, E). In addition, ELISA data showed higher serum levels of TNF-α and IL-6 in rats exposed to I/R than those of sham-operated rats. I/R-exposed rats with Dex treatment displayed a decline in the serum levels of TNF-α and IL-6 while RGFP966 treatment resulted in increased serum levels of TNF-α and IL-6 (Fig. 7F, G). Moreover, the results of IHC (Fig. 7H) indicated the presence of GFAP and S-100β in the EGCs of rat ileal tissues, and the I/R-exposed rats presented with increased positive expression of GFAP and S-100β, relative to sham-operated rats. Besides, the positive expression of GFAP and S-100β was diminished following Dex treatment, the effect of which was abolished by RGFP966 treatment (Fig. 7H).

In addition, western blot analysis showed that the protein expression of S-100β, GFAP, Bax, HDAC3, p53, p62 and cleaved caspase-3 in I/R-exposed rats, I/R-exposed rats with Dex alone or additional RGFP966 treatment was increased but LC3-II/I ratio, HDAC3 phosphorylation level and SIRT3, PINK1, Bcl-2, Atg5 and caspase-3 protein expression were reduced compared with sham-operated rats. Treatment with Dex in I/R-exposed rats resulted in an increase in LC3-II/I ratio, HDAC3 phosphorylation level and the protein expression of SIRT3, PINK1, Bcl-2, Atg5 and caspase-3, whereas, the protein expression of S-100β, GFAP, Bax, HDAC3, p62, p53, and cleaved caspase-3 was reduced, which were reversed by the additional treatment of RGFP966 (Fig. 7I). Furthermore, Dex treatment reduced the I/R-induced apoptosis rate whereas treatment of RGFP966 undermined this result (Fig. 7J, Additional file 2: Figure S2). Taken together, these lines of evidence indicate that Dex confers protection against intestinal I/R injury through SIRT3-mediated PINK1/HDAC3/p53 pathway in vivo.

## Discussion

Dex is a established therapeutic drug applicable for the treatment of intestinal I/R injury [13]. However, the molecular mechanism by which Dex affects intestinal I/R injury remains unclear. The pathophysiology of intestinal I/R injury includes mitochondrial damage and apoptosis, and therefore, thoroughly investigating potential targets against intestinal I/R injury via mitochondrial damage and apoptosis is essential in the development of therapy [49, 50]. Thus, the current study suggested that Dex attenuated the intestinal I/R injury by reducing the mitochondrial damage and apoptosis of EGCs through the SIRT3-mediated PINK1/HDAC3/p53 pathway.

The present study was conducted by using in vivo intestinal I/R injury rat model and in vitro TNF-α and IFN-γ-stimulated EGCs inflammation model. Based on a previous study, intestinal I/R injury was characterized by the presence of intestinal epithelial barrier dysfunction while EGCs were capable of conferring functional integrity of intestinal epithelial barrier dysfunction [51]. Our initial finding was suggestive of the protective effect of Dex in rats with intestinal I/R injury, accompanied by reduced oxidative stress, inflammatory response, and EGC apoptosis. Consistently, oxidative stress and inflammation have been attributed to the pathogenesis of intestinal I/R injury, while its reduction has been associated with the improvement in intestinal I/R injury [52]. Similarly, Chen et al. verified that oxidative stress and inflammatory response are inhibited by Dex in rats with lung injury during intestinal I/R injury [53].

Moreover, our results demonstrated increased LC3-II/I ratio and expression of SIRT3, PINK1, Bcl-2, and caspase-3 and decreased expression of Bax, p53, and cleaved caspase-3. Based on these results, it can be concluded that Dex promoted mitophagy, while inhibiting mitochondrial damage and apoptosis of EGCs. Consistently, Dex can upregulate the expression of autophagy and mitophagy related proteins, such as Beclin-1, LC3 II and PINK1, and thus enhances autophagy, resulting in the removal of damaged mitochondria in lipopolysaccharide (LPS)-induced acute kidney injury [54]. Meanwhile, increasing studies have confirmed that autophagy induction is essential for alleviation of intestinal I/R injury [55, 56]. Caspase-3 (a member of cysteine proteases family) demonstrates a central role in the proteolysis during apoptosis. Thus, cleaved caspase-3 is regarded as a reliable marker of apoptosis [57]. On the other hand, Bcl-2 plays an anti-apoptotic role by serving as a core regulator of the intrinsic apoptosis pathway [58]. Bax is a member of the Bcl-2 family which is considered a key step in apoptosis [59]. Notably, LC3-II is an autophagosomal marker located on the surface of the autophagy vesicle membrane and has been in the assessment of the processes related to autophagy [60]. Consistent with our results, a corroborating study suggested that Dex has ameliorative effects on intestinal I/R injury via suppression of mitochondrial apoptosis and inflammatory response [60]. Additionally, Sun et al. provided evidence suggesting that Dex treatment reduced the Bax expression in the intestinal tissues while enhancing Bcl-2 and caspase-3 expression, which is indicative of its preventive effects against intestinal I/R injury [61]. Wang et al. have also reported that sustaining mitochondrial localization of PHB2 by Bax inhibitor-1 (BI1) could serve as a novel therapeutic target for the treatment of acute kidney injury [62].

The current study further evaluated the role of SIRT3-mediated PINK1 related pathway and its correlation with ameliorative effects of Dex on intestinal I/R injury. Collectively, our finding demonstrated that Dex facilitated the TNF-α- and IFN-γ-stimulated EGCs mitophagy and impeded apoptosis via activation of SIRT3-mediated PINK1-related pathway. Das et al. revealed that the SIRT3-medicated PINK1/PRKN signaling pathway induced mitophagy [14]. Further, Wang et al. also indicated that SIRT3 relieved severe mitochondrial oxidative damage, apoptosis, and distal organ damage that occur secondary to intestinal I/R injury [63]. Moreover, a recent finding by Zhou et al. has illustrated novel therapeutic strategies that target the balance among NR4A1, fission, and mitophagy which might provide a survival advantage to microvasculature following IR stress [64]. Furthermore, activation of SIRT3 has been shown to play a major role in improving the severity of I/R injury, including myocardial and cerebral I/R injury [19, 65].The beneficial effect of downregulated expression of PINK1 in intestinal I/R injury has been previously determined, the mechanism of which involves the suppression of mitochondrial division, thereby preventing intestinal I/R injury [66]. On the other hand, the upregulation of PINK1 has been indicated to reduce the p53 expression and exert protective effects against cerebral I/R injury [67]. Consistently, it has been previously demonstrated that the silencing of PINK1 resulted in the partial inhibition of the ability of HDAC3 in regulating p53 acetylation, whereas inhibition of HDAC3 completely revoked this inhibition [21]. Notably, our data also reported that the protective effects of Dex in intestinal I/R injury were associated with the suppression of HDAC3, accompanied by an increase in p53 expression. Consistently, previous studies have indicated that the inhibition of p53 ameliorates intestinal I/R injury [25, 68].

## Conclusion

**Fig. 8:**
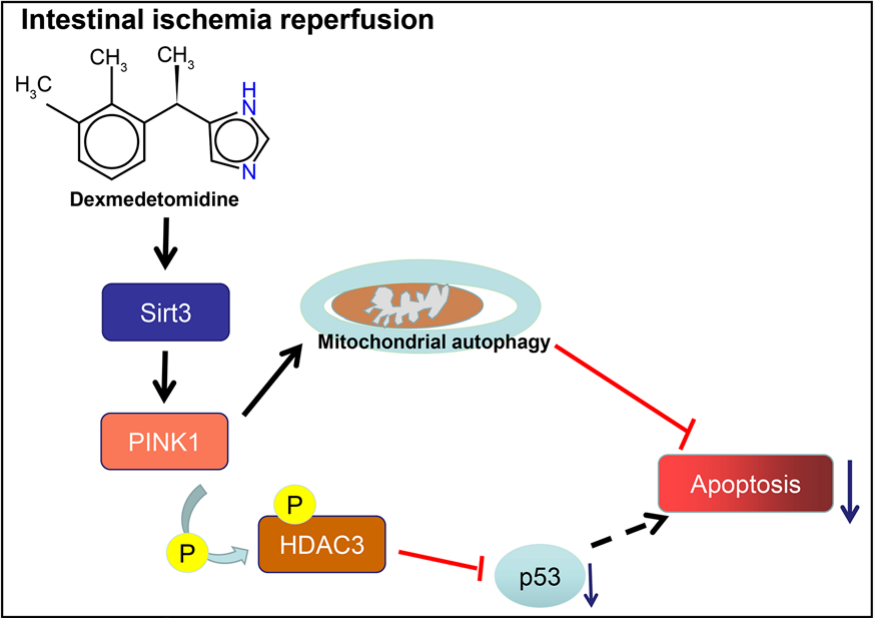
A graphic illustration of pathway representing that Dex prevents EGCs from mitochondrial damage and apoptosis by activating SIRT3-mediated PINK1/HDAC3/p53 pathway, thereby alleviating intestinal I/R injury

In summary, our findings highlighted the mechanistic actions underlying protective role of Dex against mitochondrial damage and apoptosis in EGCs through the activation of the SIRT3-mediated PINK1/HDAC3/p53 pathway, which ultimately resulted in alleviation of intestinal I/R injury (Fig. 8). Importantly, the present study revealed the mechanism underlying the function of Dex on mitochondrial damage and apoptosis, thus laying the basis for identifying novel therapeutic targets for promoting the protection Dex against intestinal I/R injury. Future studies should collect specimens from patients diagnosed with intestinal I/R injury for the in-depth analysis of the established mechanism in intestinal I/R injury to validate its applicable values in using Dex for this disease in clinical practice.

## Supplementary Information

**Additional file 1: Figure S1.** Representative images of TEM for mitophagy in TNF-α- and IFN-γ-stimulated cells in response to Dex or 3-MA (A), sh-PINK1 or PFT-α (B), Dex or 3-TYP (C), and Dex or RGFP966 (D). The yellow arrow refers to mitochondria, and the red arrow refers to mitochondrial autophagosomes. PFT-α, pifithrin-α.

**Additional file 2: Figure S2.** Representative images of TUNEL assay for the apoptosis of EGCs in intestinal tissues of rats in response to Dex or RGFP966.

**Additional file 3: Table S1.** Primer sequences for RT-qPCR.

## Data Availability

The datasets generated/analyzed during the current study are available.

## Abbreviations

I/R: Ischemia/reperfusion
EGCs: Enteric glial cells
PTEN: Phosphatase and tensin homolog
PINK1: Putative kinase 1
HDAC3: Histone deacetylase 3
SIRT3: Sirtuin 3
SD: Sprague–Dawley
SPF: Specific-pathogen free
Dex: Dexmedetomidine
SMA: Superior mesenteric artery
ELISA: Enzyme-linked immunosorbent assay
PBS: Phosphate-buffered saline
HRP: Horseradish peroxidase
IgG: Immunoglobulin G
shRNA: Short hairpin RNA
NC: Negative control
p: Phosphorylated
Bax: Bcl-2-associated X protein
Bcl-2: B-cell lymphoma 2
OD: Optical density
HE: Hematoxylin–eosin
IHC: Immunohistochemistry
BSA: Bovine serum albumin
MMP: Mitochondrial membrane potential
MDA/SOD: Malondialdehyde/superoxide dismutase
BCA: Bicinchoninic acid
FITC: Fluorescein isothiocyanate
PI: Propidium iodide
HEPES: Hydroxyethyl-piperazine-*N*′-2-ethane sulfonic acid
TEM: Transmission electron microscope
RPMI: Roswell Park Memorial Institute
FBS: Fetal bovine serum
RIPA: Radio-immunoprecipitation assay
SDS-PAGE: Sodium dodecyl sulfate-polyacrylamide gel electrophoresis
PVDF: Polyvinylidene fluoride
GAPDH: Glyceraldehyde-3-phosphate dehydrogenase
Co-IP: Co-immunoprecipitation
EDTA: Ethylenediaminetetraacetate
ANOVA: Analysis of variance
