# Supplementary figures and images for: Dexmedetomidine inhibits mitochondria damage and apoptosis of enteric glial cells in experimental intestinal ischemia/reperfusion injury via SIRT3-dependent PINK1/HDAC3/p53 pathway
Source: J Transl Med. 2021 Nov 12;19:463. doi: 10.1186/s12967-021-03027-6 (PMC8588684; doi:10.1186/s12967-021-03027-6)

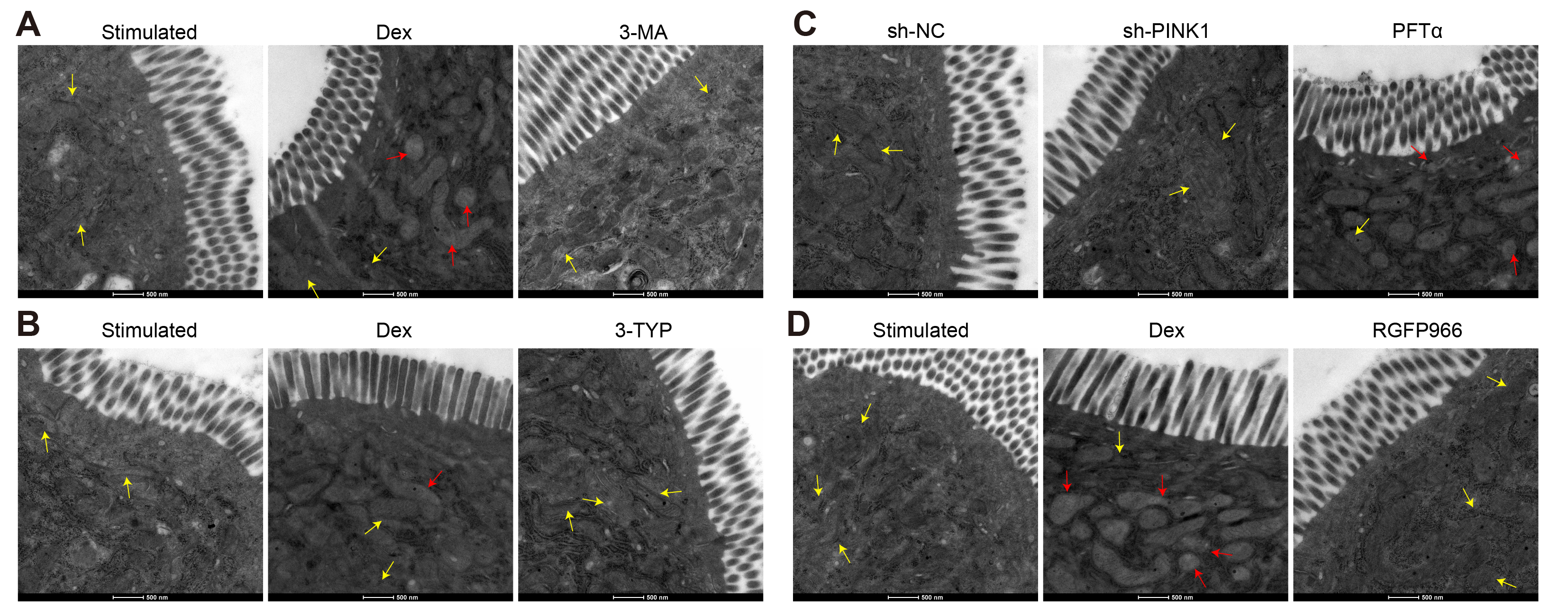

Supplement: Supplementary file 1 — Additional file 1: Figure S1. Representative images of TEM for mitophagy in TNF-α- and IFN-γ-stimulated cells in response to Dex or 3-MA (A), sh-PINK1 or PFT-α (B), Dex or 3-TYP (C), and Dex or RGFP966 (D). The yellow arrow refers to mitochondria, and the red arrow refers to mitochondrial autophagosomes. PFT-α, pifithrin-α. [file 12967_2021_3027_MOESM1_ESM.jpg]

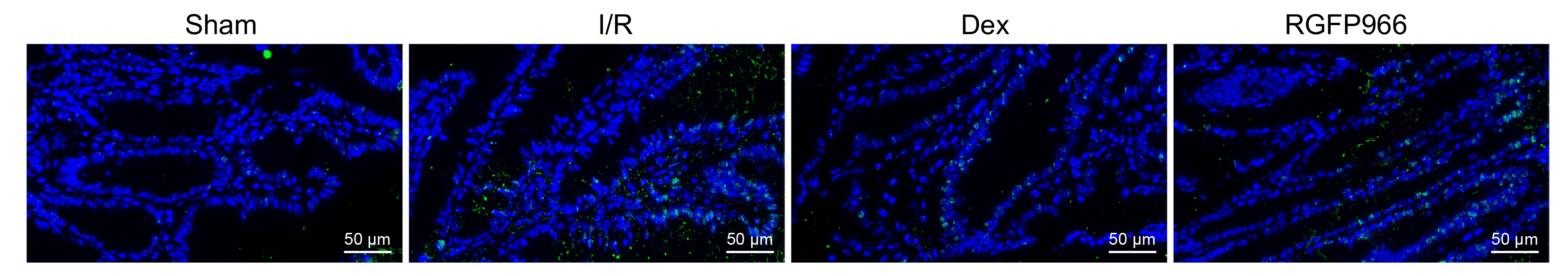

Supplement: Supplementary file 2 — Additional file 2: Figure S2. Representative images of TUNEL assay for the apoptosis of EGCs in intestinal tissues of rats in response to Dex or RGFP966. [file 12967_2021_3027_MOESM2_ESM.jpg]
