# Supplementary material for: Dexmedetomidine inhibits mitochondria damage and apoptosis of enteric glial cells in experimental intestinal ischemia/reperfusion injury via SIRT3-dependent PINK1/HDAC3/p53 pathway
Source: J Transl Med. 2021 Nov 12;19:463. doi: 10.1186/s12967-021-03027-6 (PMC8588684; doi:10.1186/s12967-021-03027-6)
Supplement: Supplementary file 3 — Additional file 3: Table S1. Primer sequences for RT-qPCR. [file 12967_2021_3027_MOESM3_ESM.docx]

**Supplementary Table 1.** Primer sequences for RT-qPCR

| Gene | Primer sequence (5’-3’) |
| --- | --- |
| PINK1-1 | F: TGTGTCGTGATGGTCTGTGA |
|  | R: CCAGCTTGGCCATTCACTTT |
| PINK1-2 | F: TACCAGTGCACCAGGAGAAG |
|  | R: GCTTGGGACCTCTCTTGGAT |
| PINK1-3 | F: GGGTCAGCACGTTCAGTTAC |
|  | R: AGAGGCTAGTCAGGAGGGAA |
| GAPDH | F: ATCAAGAAGGTGGTGAAGCA |
|  | R: AAGGTGGAAGAATGGGAGTTG |

### Note: RT-qPCR, reverse transcription quantitative polymerase chain reaction; PINK1, phosphatase and tensin homolog (PTEN)-induced putative kinase 1; GAPDH, glyceraldehyde-3-phosphate dehydrogenase; F, forward; R, reverse.
